# Concordance among methods of nutritional assessment in patients included on the waiting list for liver transplantation

**DOI:** 10.1016/j.je.2016.09.011

**Published:** 2017-05-23

**Authors:** María Teresa García-Rodríguez, Beatriz López-Calviño, María del Carmen Piñón-Villar, Alejandra Otero-Ferreiro, Francisco Suárez-López, Manuel Gómez-Gutiérrez, Sonia Pértega-Díaz, María Teresa Seoane-Pillado, Salvador Pita-Fernández

**Affiliations:** a Digestive Service, Instituto de Investigación Biomédica de A Coruña (INIBIC), Complexo Hospitalario Universitario de A Coruña (CHUAC), SERGAS, Universidade da Coruña, A Coruña, Spain; b Clinical Epidemiology and Biostatistics Research Group, Instituto de Investigación Biomédica de A Coruña (INIBIC), Complexo Hospitalario Universitario de A Coruña (CHUAC), SERGAS, Universidade da Coruña, A Coruña, Spain; c Transplant Coordination, Instituto de Investigación Biomédica de A Coruña (INIBIC), Complexo Hospitalario Universitario A Coruña (CHUAC), SERGAS, Universidade da Coruña, A Coruña, Spain

**Keywords:** Malnutrition, Liver transplantation, Validity, Diagnostic accuracy, Concordance

## Abstract

**Background:**

The aim of the present study was to determine the extent of malnutrition in patients waiting for a liver transplant. The agreement among the methods of nutritional assessment and their diagnostic validity were evaluated.

**Methods:**

Patients on the waiting list for liver transplantation (n = 110) were studied. The variables were: body mass index, analytical parameters, liver disease etiology, and complications. Liver dysfunction was evaluated using the Child–Pugh Scale. Nutritional state was studied using the Controlling Nutritional Status (CONUT), the Spanish Society of Parenteral and Enteral Nutrition (SENPE) criteria, the Nutritional Risk Index (NRI), the Prognostic Nutritional Index (PNI-O), and the Subjective Global Assessment (SGA). Agreement was determined using the Kappa index. Area under receiver operator characteristic curves (AUCs), the Youden index (J), and likelihood ratios were computed.

**Results:**

Malnutrition varied depending on the method of evaluation. The highest value was detected using the CONUT (90.9%) and the lowest using the SGA (50.9%). The pairwise agreement among the methods ranged from K = 0.041 to K = 0.826, with an overall agreement of each criteria with the remaining methods between K = 0.093 and K = 0.364. PNI-O was the method with the highest overall agreement. Taking this level of agreement into account, we chose the PNI-O as a benchmark method of comparison. The highest positive likelihood ratio for the diagnosis of malnutrition was obtained from the Nutritional Risk Index (13.56).

**Conclusions:**

Malnutrition prevalence is high and prevalence estimates vary according the method used, with low concordance among methods. PNI-O and NRI are the most consistent methods to identify malnutrition in these patients.

## Highlights

• Malnutrition prevalence in patients waiting for a liver transplant is high.
• The prevalence of malnutrition varied depending on the method of evaluation.
• The overall agreement among the malnutrition assessment methods was low.
• PNI-O was the method of assessment malnutrition with the highest overall agreement.
• NRI obtained the highest positive likelihood ratio for the diagnosis of malnutrition.

## Introduction

The presence of protein-calorie malnutrition is common among patients with liver disease. Malnutrition is one of many possible effects of liver disease.^1^, ^2^, ^3^ This malnutrition is associated with the degree of hepatic dysfunction and increased morbidity both before and after transplantation.^4^, ^5^, ^6^, ^7^

Great variability in the prevalence of malnutrition has been observed, depending on the method used for assessment^5^ and on the severity of the disease, which can change the body composition and analytical parameters.^6^ Therefore, there is no consensus among authors on which are the most effective methods to assess the nutritional state of these patients.

Because there is not a gold standard, different tools must be used to quantify and classify malnutrition.^2^, ^8^ The most frequently used methods to assess the nutritional state in cirrhotic patients include anthropometric parameters and analytical parameters.^1^, ^2^, ^5^ Other methods are the Subjective Global Assessment (SGA),^1^, ^9^ handgrip strength,^6^, ^10^ bioelectrical impedance analysis,^11^ or carrying out dietary assessments using tools such as the Malnutrition Universal Screening Tool (MUST) or Nutritional Risk Screening 2002 (NRS-2002).^4^

The European Society for Clinical Nutrition and Metabolism recommends evaluating malnutrition in patients with liver cirrhosis through tests, including subjective global assessment, anthropometry, biomedical impedance, and grip strength evaluation.^12^ The American Society for Parenteral and Enteral Nutrition advises performing a nutritional screening using the Mastrich Index, Nutritional Risk Index (NRI), and Prognostic Nutritional Index or Index Onodera (PNI-O), among other tests, in the first 24 h after hospital admission for all adult patients. Furthermore, malnourished patients should be assessed using the Mini Nutritional Assessment (MNA) and SGA.^13^ The Spanish Society of Parenteral and Enteral Nutrition (SENPE) recommends simple and understandable nutritional assessment methods that include clinical and laboratory parameters in all hospital-admitted adult patients.^14^ SENPE criteria include anthropometric parameters (body mass index [BMI] and weight loss) and laboratory values (albumin, cholesterol, and lymphocytes).

Following the recommendations and due to the variability among methods, some authors have tried to identify the best tool to assess the nutritional state of these patients. Villalobos et al^15^ created a screening method based on the recommendations of the SENPE and compared it with other methods. Taniguchi et al^16^ demonstrated that the PNI-O and Controlling Nutritional Status (CONUT) are useful tools to assess the nutritional state of patients with chronic liver disease. Finally, Fernandes et al^11^ assessed the nutritional status of patients with cirrhosis through anthropometric measurements, the SGA, hand grip strength, and bioelectrical impedance. The aim of their study was to identify the safest and most effective method for the assessment of the nutritional state.

This study was performed due to the significance of the nutritional state on morbidity and mortality of patients and the difficulty in determining a nutritional assessment method. We aimed to determine the malnutrition degree in patients on the liver transplant list, study the concordance among assessment methods, and explore the diagnostic validity compared to a benchmark that was chosen because it obtained the best overall concordance.

## Methods

### Setting

We conducted a cross-sectional study at University Hospital Complex in A Coruña, Spain performed during the period of January 2012 through December 2014.

### Sampling and inclusion criteria

The inclusion criteria were patients over the age of 18 years who were included on the waiting list for liver transplant during the study period and who signed the informed consent.

### Sample size calculation

Sample size was limited by both the duration of the study and the number of liver transplants per year. We evaluated 110 patients during the period of this study (2012–2014).

This sample size (n = 110) allow us to estimate characteristics related to nutritional state with a precision of ±10% and 95% confidence (α = 0.05), assuming a prevalence of 50% of the variable of interest and 10% losses during follow-up. At the same time, this sample size allows estimation of the pairwise agreement among methods with a precision of ±16.50% and 95% confidence, assuming a Kappa index value around 0.400.

### Data collection and measurements

The patients were identified with a code, keeping their personal data confidential. To obtain the necessary information for the study, clinical records were reviewed, a physical examination was performed, and the patients were interviewed.^17^

The following variables were obtained by the time of inclusion on the waiting list for liver transplantation:

1. From clinical records: demographic data (age and gender), analytical parameters (albumin, cholesterol, total lymphocyte, bilirubin, creatinine, and international normalize ratio), transplant etiology (alcoholic cirrhosis, autoimmune hepatitis, polycystic disease, liver cancer, primary biliary cirrhosis, and viral cirrhosis) and the presence of hepatic complications (ascites, hepatic encephalopathy, digestive bleeding, bacterial peritonitis, and hepatorenal syndrome)
2. From physical examination: patient weight, height, and BMI
3. From interviews: the SGA^18^

The degree of liver dysfunction was evaluated using the Child–Pugh Scale,^19^, ^20^ in which higher score indicate greater liver dysfunction. The assessment of nutritional state was performed using validated scales (Table 1): CONUT,^21^ SENPE criteria,^14^, ^22^ NRI,^8^, ^23^ and PNI-O.^16^

**Table 1:** Nutritional assessment by different methods.

Methods (Reference number)	Description	Malnutrition classification
CONUT^21^	Gives a score to values of *albumin*, *cholesterol*, and *total lymphocytes*, getting a total score among 0–12 points	Scores ≥2 points
SENPE criteria^8^, ^14^, ^22^	Based on three criteria: A criteria: *Weight loss* >5% in 1 month or >10% in 6 months or *BMI* <18 kg/m^2^ B criteria: *Albumin* <3.5 g/dL C criteria: *Total lymphocyte* <1600 c/mm^3^ and/or *Cholesterol* <180 mg/dL	Meet 2 of 3 criteria
NRI^8^, ^23^	1.519 × *serum albumin* (g/L) + 41.7 × (*current weight*/*usual weight*)	Values ≤ 100 points
PNI-O^16^	10 × *serum albumin* (g/dL) + 0.005 × *Total lymphocyte* (cells/mm^2^)	Values < 40 points
SGA^18^	Consists of two parts, medical history and physical examination.	**Moderate:** reduced intake, with functional changes and the change in body mass absent or scarce **Severe:** evident decreases in food intake and body mass function

CONUT, Controlling Nutritional Status; NRI, Nutritional Risk Index; PNI-O, Prognostic Nutritional Index or Onodera Index; SENPE criteria, Spanish Society of Parenteral and Enteral Nutrition criteria; SGA, The Subjective Global Assessment.

The CONUT and PNI-O scores were selected because they have been shown to be useful tools to assess the nutritional state in patients with chronic liver disease.^16^ Two other method, the NRI and SENPE recommendations, have been selected based on the recommendations of international and national associations.

Finally, the SGA^18^ was also performed, which consists of two parts: a review of clinical records (changes in weight and intake, gastrointestinal symptoms, functional capacity, and underlying disease) and a physical exam to assess the loss of fat-mass muscle, ascites or edema, and the presence of tongue or skin lesions. Patients were classified as well nourished, moderately malnourished or at risk of malnutrition, and severely malnourished. The SGA has been the preferred nutritional evaluation method for liver transplantation candidates.

Although some of these methods could be considered as nutritional screening tools (CONUT, NRI, and PNI-O), while others could be considered as assessment tools (SGA), all of them allow us to calculate the risk of malnutrition.

### Statistical analysis

Descriptive analysis was performed for all variables. Continuous variables were reported using means and standard deviations (SD) or medians and interquartile ranges, as appropriate. For dichotomous/categorical variables, absolute numbers and percentages were computed, together with their 95% confidence intervals.

Concordance between the different scales that assess nutritional state was studied with the statistical Kappa (K) index. The overall Kappa was estimated, and the homogeneity test was performed to study the agreement amongst methods.

PNI-O was the method of the highest level of overall agreement. Taking this level of agreement into account, we chose the PNI-O as a benchmark method of comparison.

Diagnostic validity was assessed using the area under the curves (AUCs), the Youden index, sensitivity, specificity, positive and negative predictive values, and positive and negative likelihood ratios. The results of the CONUT and the NRI were compared with PNI-O diagnosis of malnutrition as the reference criteria and displayed on a receiver operating characteristic (ROC) curve. For each method, the ROC AUC was computed, and its optimum cut-off point was determined using the Youden index. The Youden index (J) is defined as the maximum vertical distance between the ROC curve and the diagonal or change line and calculated as J = max (sensitivity + specificity − 1).^24^, ^25^ Using the cut-off points determined by assessing the ROC curves, the positive and negative predictive values of the diagnostic tests were calculated. In order to determine the diagnostic accuracy of the tests, their likelihood ratios were calculated.^26^ Statistical analysis was performed using SPSS for Windows (version 19.0, SPSS Inc., Chicago, IL, USA) and Epidat version 3.1 (Dirección Xeral de Innovación e Xestión da Saúde Pública, Xunta de Galicia y Organización Panamericana de la Salud, La Coruña, Spain).

### Ethics

The study complies with the principles laid down in the Declaration of Helsinki. Informed consent was obtained from all the participants in the study. Confidentiality was preserved in accordance with the current Spanish Data Protection Law (15/1999). This project was approved by the corresponding ethics review board (Clinical Research Ethical Committee of Galicia, #2010/081).

## Results

The mean age of our patients was 56.85 (SD, 8.23) years, and most were men (72.73%). The BMI was ≥25 kg/m^2^ in 58.2% of them. Mean Child–Pugh score was 8.37 (SD, 2.12), and 48.6% of the patients showed a significant functional commitment (Child–Pugh B). The main etiology was alcoholic cirrhosis (52.7%). The most frequent liver disease complication was ascites (79.1%) (Table 2).

**Table 2:** Baseline characteristics of the study sample previous to liver transplant.

Variables	n	Mean (SD)	95% CI
**Age,** years	110	56.85 (8.23)	(55.30–58.41)
**BMI,** kg/m^2^	110	26.99 (5.05)	(26.04–27.95)
**Child–Pugh**	110	8.37 (2.12)	(7.97–8.77)
	**n**	**%**	**95% CI**
**Gender**			
Male	80	72.73	(63.95–81.51)
**BMI categories**			
Normal weight	46	41.82	(32.15–51.49)
Overweight	37	33.64	(24.35–42.92)
Obese	27	24.55	(16.05–33.04)
**Child–Pugh categories**			
A	24	22.00	(13.65–29.99)
B	53	48.60	(38.39–57.97)
C	32	29.40	(20.15–38.03)
**Liver disease etiology**			
Alcoholic cirrhosis	58	52.73	(42.94–62.51)
Viral cirrhosis	34	30.91	(21.82–40.00)
Primary biliary cirrhosis	5	4.54	(1.50–10.30)
Liver tumor	34	30.91	(21.82–40.00)
Polycystic liver disease	1	0.91	(0.02–4.96)
Autoimmune hepatitis	10	9.10	(3.30–14.91)
Other	13	11.82	(5.33–18.31)
**Complications**			
Ascites	87	79.10	(71.04–87.15)
Encephalopathy	67	69.90	(51.34–70.50)
Digestive hemorrhage	29	26.36	(17.68–35.05)
Bacterial peritonitis	12	10.91	(4.63–17.19)
Liver-kidney syndrome	21	19.10	(11.29–26.89)
Portal hypertension	75	68.18	(59.02–77.34)

CI, confidence interval; BMI, body mass index; SD, standard deviation.

### Prevalence of malnutrition according to the degree of liver dysfunction

**Fig. 1:**
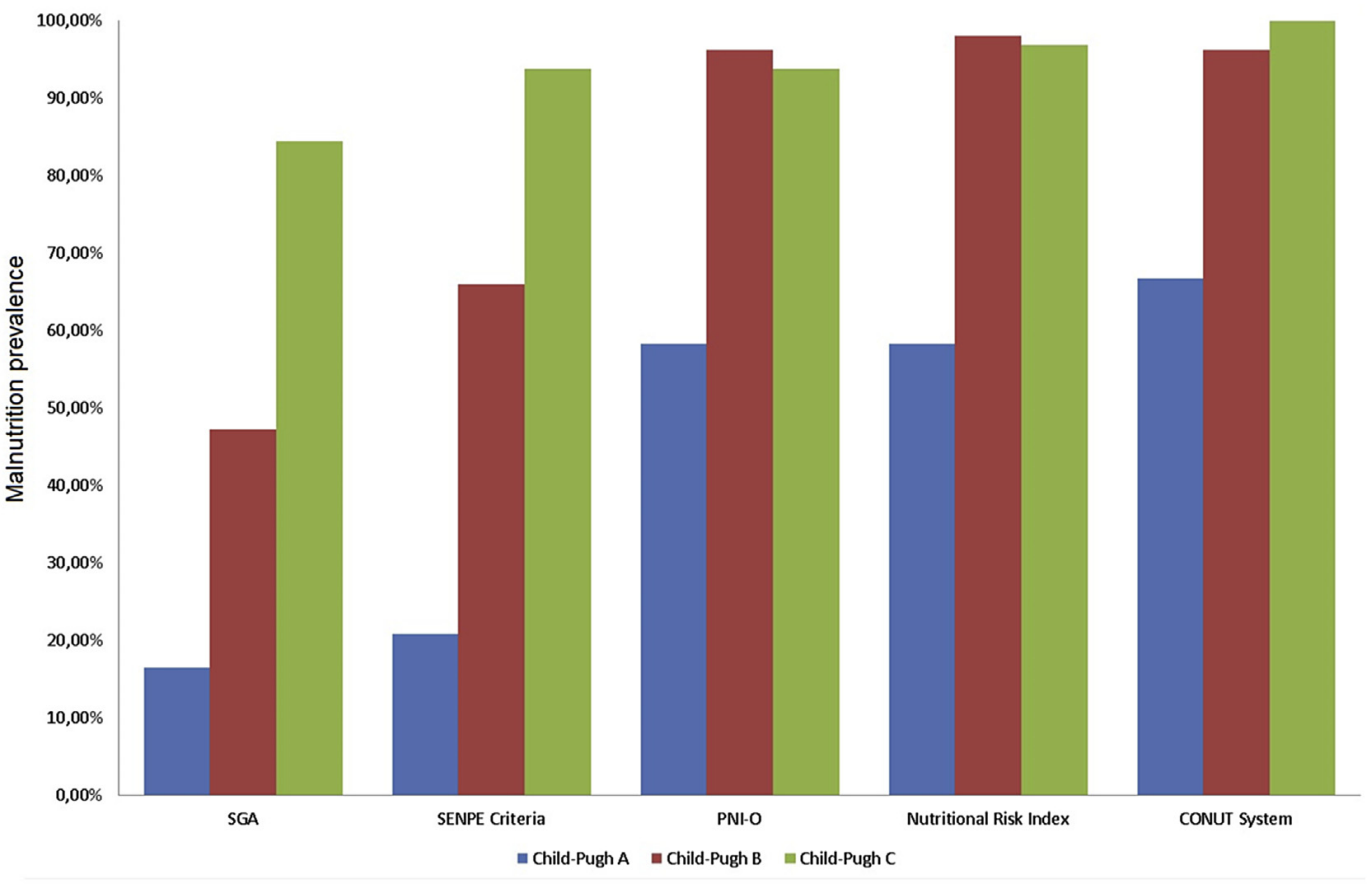
Distribution of malnutrition prevalence according with the degree of hepatic dysfunction (Child–Pugh).

The percentage of malnutrition at the time of inclusion in the transplant waiting list (Table 3) changed depending on the method of evaluation. The highest prevalence of malnutrition was estimated using the CONUT (90.9%), and the lowest prevalence was estimated using the SGA (50.9%). Examining the prevalence of malnutrition according to the Child–Pugh classification, we found that the higher the hepatic dysfunction, the worse the nutritional state (p < 0.001) (Fig. 1).

**Table 3:** Prevalence of malnutrition previous liver transplant.

Malnutrition		n	%	95% CI
**NRI**				
Nourished		12	10.90	(4.63–17.19)
Undernourished	Mild malnutrition	6	5.46	(0.76–10.15)
	Moderated malnutrition	54	49.09	(39.29–58.89)
	Severe malnutrition	38	34.55	(25.21–43.89)
**SENPE criteria**				
Nourished		39	35.46	(26.06–44.85)
Undernourished		71	64.55	(55.15–73.94)
**CONUT system**				
Nourished		10	9.10	(3.26–14.92)
Undernourished	Mild malnutrition	38	34.50	(33.91–53.36)
	Moderated malnutrition	42	38.20	(28.65–47.72)
	Severe malnutrition	20	18.20	(10.52–25.84)
**SGA Test**				
Nourished		54	49.09	(39.29–58.89)
Undernourished	Moderated malnutrition	49	44.55	(34.80–54.29)
	Severe malnutrition	7	6.36	(1.35–11.38)
**PNI-O**				
Nourished (PNIO ≥40)		14	12.73	(6.05–19.41)
Undernourished (PNIO <40)		96	87.27	(80.59–93.96)

CI, confidence interval; CONUT, Controlling Nutritional Status; NRI, Nutritional Risk Index; PNI-O, Prognostic Nutritional Index or Onodera Index; SENPE, Spanish Society of Parenteral and Enteral Nutrition; SGA, The Subjective Global Assessment.

### Agreement between methods of nutritional assessment

Due to the variability in the prevalence of malnutrition depending on the method used, we calculated the level of agreement among different methods. Pairwise agreement among the methods ranged from K = 0.041 to K = 0.826, with an overall agreement of each criteria with the remaining methods between K = 0.093 and K = 0.364 (Table 4). PNI-O was the method with the highest level of overall agreement. Taking this level of agreement into account, we choose the PNI-O as our reference criterion to study the diagnostic accuracy with the rest of the methods.

**Table 4:** Prevalence of malnutrition and level of agreement with different methods.

Prevalence of malnutrition	CONUT	NRI	PNI-O	SENPE criteria	SGA
	90.91%	89.09%	87.27%	64.55%	50.91%
**Kappa Index among methods**					
**Methods**	**CONUT**	**NRI**	**PNI-O**	**SENPE criteria**	**SGA**
CONUT	1	0.495	0.441	0.308	0.114
NRI		1	0.826	0.365	0.041
PNI-O			1	0.420	0.042
SENPE criteria				1	0.251
Overall Agreement	K = 0.232; p = 0.010	K = 0.334; p < 0.001	K = 0.364; p < 0.001	K = 0.338; p = 0.54	K = 0.093; p = 0.20

CONUT, Controlling Nutritional Status; NRI, Nutritional Risk Index; PNI-O, Prognostic Nutritional Index or Onodera Index; SENPE, Spanish Society of Parenteral and Enteral Nutrition; SGA, The Subjective Global Assessment.

### Diagnostic accuracy to identify malnutrition compared to the PNI-O (reference criterion)

The Youden index, corresponding cut-off points, sensitivity, specificity, positive and negative predictive values, and positive and negative likelihood ratios are shown in Table 5. According to the values of Youden Index, the most accurate diagnostic method for malnutrition was the NRI, followed by SENPE and CONUT.

**Table 5:** Diagnostic accuracy from different methods using PNI-O as benchmark.

	NRI		CONUT		SENPE criteria		SGA	
	Value	95% CI	Value	95% CI	Value	95% CI	Value	95% CI
Youden Index	0.96	(0.76–1.04)	0.54	(0.30–0.77)	0.74	(0.65–0.83)	0.09	(−0.19 to 0.37)
Cutt-off point	98.39	–	3.50	–	–	–	–	–
AUC	0.987	(0.970–1.00)	0.847	(0.749–0.946)	–	–	–	–
Sensitivity	96.88	(90.48–99.19)	75.00	(64.93–83.03)	100.00	(96.43–100.00)	57.14	(27.65–86.64)
Specificity	92.86	(64.17–99.63)	78.57	(48.82–94.29)	73.96	(64.66–83.26)	52.08	(41.57–62.60)
Positive Predictive Value	98.94	(93.38–99.94)	96.00	(87.97–98.96)	35.90	(19.56–52.23)	14.81	(4.41–25.12)
Negative Predictive Value	81.25	(53.69–95.03)	31.43	(17.43–49.42)	100.00	(99.30–100.00)	89.29	(80.29–98.28)
Prevalence of malnutrition	89.09	(82.81–95.37)	90.91	(85.08–96.74)	64.55	(55.15–73.94)	50.91	(41.11–60.71)
Positive Likelihood Ratio	13.56	(2.05–89.69)	3.50	(1.28–9.61)	3.84	(2.74–5.38)	1.19	(0.72–1.96)
Negative Likelihood Ratio	0.03	(0.01–0.10)	0.32	(0.20–0.49)	–	–	0.82	(0.44–1.55)

AUC, area under the curve; CI, confidence interval; CONUT, Controlling Nutritional Status; NRI, Nutritional Risk Index; SENPE, Spanish Society of Parenteral and Enteral Nutrition Criteria; SGA, Subjective Global Assessment.

**Fig. 2:**
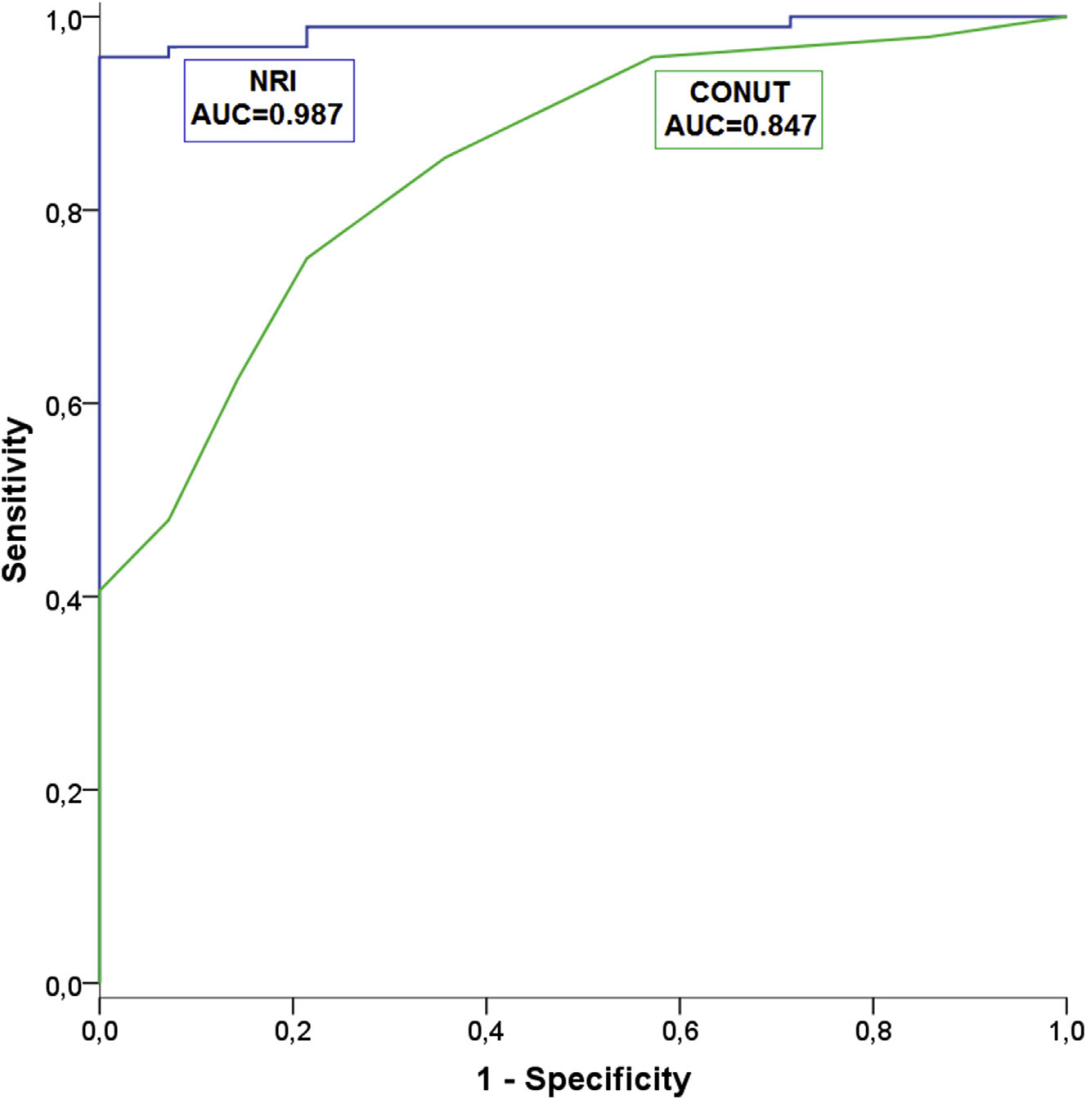
Receiver operating characteristic curve for different scores to the diagnoses a malnutrition using PNI-O as benchmark.

Using the NRI and the CONUT (quantitative methods), the cutoff point for malnutrition in patients with liver disease was found to be higher compared to the general cutoff point. The ROC curves for the methods of malnutrition analysis are displayed in Fig. 2. The AUC showed that the NRI was most accurate in predicting malnutrition (AUC, 0.987), followed by the CONUT (AUC, 0.847), which was moderately accurate.

From the ROC curves, the optimal cut-off points of these tests for diagnosing malnutrition in the total sample were 98.39 for the NRI and 3.50 for the CONUT, with sensitivities of 96.9% and 75.0%, respectively. The positive predictive values for the NRI and the CONUT were 98.9% and 96.0%, respectively. Negative predictive values were 81.3% and 31.4%, respectively. The NRI was found to have the highest positive likelihood ratio (13.56).

## Discussion

### Prevalence of malnutrition

It is important to know the nutritional state of the patient, as it is a risk factor for morbidity and correlates with the severity of liver dysfunction,^27^ both before and after transplantation.^5^, ^7^ To the alteration in analytical parameters due to the disease,^2^, ^3^ the variability in the malnutrition prevalence is added,^2^, ^8^, ^17^ as shown in the literature.^16^, ^17^

Among the different evaluation methods, anthropometric parameters, such as BMI, are used. In this study, there were no patients with malnutrition according to BMI, and more than half of the subjects were classified as overweight or obese. This result may be due to most of the patients having ascites, which involves a change in body composition. This finding is similar to those of other publications, which conclude that BMI underestimates malnutrition and is not a suitable method to evaluate these patients.^4^, ^16^ In the study by Villalobos et al, 5% of hospitalized patients (regardless of pathology) were classified with possible malnutrition according to BMI, a value which is very low in relation to those estimated using other evaluation methods. In conclusion, as BMI is a late indicator of malnutrition,^15^ it was not included in this study to assess concordance or diagnostic validity.^16^, ^17^

The methods used in this study can be distributed into three groups: a) structured tests (e.g., the SGA); b) methods using only analytical parameters (CONUT and PNI-O); and c) methods using analytical and anthropometric methods (SENPE criteria and the NRI). The SGA and SENPE criteria showed lower prevalence estimates of malnourished patients (50.9% and 64.6%, respectively). The low prevalence of malnutrition estimated using the SGA is similar to that reported in other publications,^10^, ^11^, ^16^ and this may be due to the subjective nature of this method, which underestimates malnutrition in these patients.^28^ Tanaguchi et al believe that, although the SGA is effective to assess the nutritional state in patients with other diseases, it is not a suitable screening tool for the nutritional evaluation of patients with liver disease.^28^

We found a similar prevalence of malnutrition using the SENPE criteria to that reported by Villalobos et al.^15^ The results can be related to anthropometric parameters, such as BMI and weight loss. These anthropometric parameters are affected by the possible presence of hydropic decompensation, and most of our patients had ascites.

The NRI, CONUT, and the PNI-O all showed higher malnutrition prevalence (89.1%, 90.9% and 87.3%, respectively) than SENPE criteria and SGA. This can be explained by the pathology of patients, which affects the values of albumin, cholesterol (which is synthesized by the liver), and lymphocytes (a parameter related to protein depletion and a malnutrition indicator). In addition, in this study, patients with mild malnutrition were considered malnourished, while only moderate and severe malnutrition patients were considered malnourished in other publications.^8^, ^15^, ^16^ The association between degree of liver dysfunction and malnutrition is consistent with other publications.^16^

### Concordance of nutritional methods

This study shows that the concordance among the methods of nutritional assessment is not very high (Table 2). The concordance obtained between the SGA and CONUT was low, while it was moderate in studies by Ulibarri et al and Hernandez-Escalante et al (K = 0.488 and K = 0.677, respectively).^21^, ^29^

The concordance between the CONUT and the NRI was moderate and similar to the findings of Gimeno et al (K = 0.547). But the results of SENPE criteria with the NRI and the CONUT were weak for both of them and lower than the results obtained by Gimeno et al, with K values of 0.609 and 0.593, respectively.^8^

Our results show a lower concordance than that reported in other publications. These findings may be related to the inclusion criteria, because we included patients with liver cirrhosis who were not considered in other published articles.^8^, ^21^, ^29^

In methods that also include anthropometric parameters (SENPE and NRI), a lower prevalence of malnutrition was observed in comparison with the CONUT and PNI-O because the increase of weight is considered as an indicator of improved nutritional state. This situation is not realistic in our sample because most of the patients had hydropic decompensation (ascites and/or edema).

The method that showed the lowest level of agreement was SGA. This is reasonable, since it is the only method of those considered that does not include anthropometric and/or laboratory parameters. Furthermore, other authors have also demonstrated that SGA is not sufficient as a nutritional screening tool for patients with liver diseases,^28^ arguing that SGA does not include any component to assess the capacity of hepatic metabolism. This is the reason that SGA could underestimate malnutrition prevalence.

The overall Kappa values, from highest to lowest, were for the PNI-O, the SENPE criteria, the NRI, and the CONUT. We decided to use the PNI-O as benchmark to determine the diagnostic validity of the other assessment methods because it showed the highest concordance.

### Diagnostic validity of the methods in comparison with the PNI-O

Although this study was not designed to identify a gold standard for malnutrition assessment in liver disease patients, we explored the results obtained to determine the diagnostic validity of each of the methods considered with respect to the method that showed the highest consistency (the PNI-O). Although the existence of reference bias cannot be ruled out, the results could be useful to identify suitable methods of assessing malnutrition in these patients.

Using the PNI-O as the reference method, the methods that showed a higher diagnostic validity for malnutrition according to the Youden Index were the NRI, SENPE criteria, and CONUT. The methods that can be considered good tools to identify malnutrition, due to having high sensitivity, are the SENPE criteria and the NRI (sensitivities of 100% and 96.9%, respectively). The methods that best identify the nourished patients, due to having high specificity, are the NRI and the CONUT (specificities of 92.9% and 78.6%, respectively).

Likelihood ratios were calculated to assess the accuracy and validity of the diagnostic test and to aid in selecting an appropriate diagnostic test. Moreover, this statistic has advantages over sensitivity and specificity because it is less likely to change with the prevalence of diagnosis.

The highest positive likelihood ratio (LR) for the diagnosis of malnutrition was obtained from the NRI (13.56). According with the level of evidence of Canadian Evidence-Based Medicine, when a test has a positive LR > 10, it is considered a good test to confirm the diagnosis of malnutrition.^30^

These findings demonstrate that the NRI is highly accurate in the diagnosis of undernutrition in our sample, being the method with the best agreement with the PNI-O, with a value Kappa of 0.846.

## Conclusions

The present study found high prevalence of malnutrition among patients on the waitlist for a liver transplant and variability in the estimated prevalence of malnutrition depending on the method of evaluation. Moreover, the nutritional assessment methods do not show good concordance. The most valid methods for identifying malnutrition in patients with liver disease are the PNI-O and the NRI.

## Conflicts of interest

None declared.

## Authors' contributions to manuscript

MTGR, MCPV, BLC and SPF participated in the design and coordination of the study. BLC, SPD and MTSP are the biostatistician of the study. MTGR, MCPV, BLC, AOF, FSL, MGG and SPF reviewed the study protocol and made suggestions that improved the design. All of these individuals are involved in the management of the study. MTGR and MCPV obtained the data for the study.

MTGR, MCPV, BLC and SPF drafted the manuscript. All the authors read, revised, and approved the final manuscript.
